# Revisiting the Link Between Factor XII and Recurrent Pregnancy Loss: A Scoping Review

**DOI:** 10.1111/aji.70127

**Published:** 2025-08-04

**Authors:** Emma Bundgaard, Malene Kræpping Pedersen, Pinar Bor, Mustafa Vakur Bor

**Affiliations:** ^1^ Department of Gynecology and Obstetrics Aarhus University Hospital Aarhus Denmark; ^2^ Department of Psychiatry Regional Hospital Viborg Viborg Denmark; ^3^ Clinical Medicine Aarhus University Aarhus Denmark; ^4^ Department of Clinical Biochemistry University Hospital of Southern Denmark Esbjerg Denmark; ^5^ Unit for Thrombosis Research, Department of Regional Health Research University of Southern Denmark Esbjerg Denmark

**Keywords:** coagulation, contact activation system, factor XII, factor XII antibody, miscarriage, recurrent pregnancy loss

## Abstract

Pregnancy loss affects approximately 23% of women, with 1%–3% experiencing recurrent pregnancy loss (RPL), defined as two or more consecutive miscarriages. Despite extensive research, up to 50% of RPL cases remain unexplained, making it a complex issue in reproductive medicine. Coagulation Factor XII (FXII) a key component of the contact activation pathway, has been suggested to play a role in RPL. Low FXII levels may lead to placental dysfunction and hypercoagulability, increasing the risk of adverse pregnancy outcomes, including RPL. This scoping review aimed to evaluate the association between FXII levels and RPL. Literature was retrieved from the PubMed database and EMBASE by a systematic search. A total of 218 studies were identified, of which 12 met the inclusion criteria published between 1992 and 2015, encompassing a total of 2362 RPL patients. They investigated FXII activity (*n* = 7), the C46T polymorphism (*n* = 3), or autoantibodies to FXII (*n* = 2) association to RPL. Available evidence suggests a potential association between low FXII levels, FXII antibodies, and RPL, probably via a prothrombotic mechanism as indicated by studies conducted 10–30 years ago. This highlights the need for further and more recent research to better elucidate the role of FXII in reproductive health and pregnancy outcomes.

## 1 Introduction

A pregnancy loss (miscarriage) is defined as the spontaneous demise of a pregnancy before the fetus reaches viability. The term includes all pregnancy losses from the time of conception until 24 weeks of gestation [1]. Recurrent pregnancy loss (RPL) is defined differently, some define RPL as two or more consecutive failed clinical pregnancies confirmed by ultrasound or histopathology other by three or more [2, 3].

RPL is classified into primary and secondary RPL. Primary RPL refers to pregnancy loss in women who have never had a live birth, whereas secondary RPL occurs in women with a history of at least one live birth [4]. The prevalence of RPL is varying from 0.8% to 3% [1] depending on study definitions and population selection, which contribute to inconsistencies in reported prevalence rates.

Several major factors contribute to RPL, including chromosomal abnormalities, anatomical structural irregularities, infections, immune system dysregulation, and endocrine disorders. However, in approximately 50% of cases the underlying cause remains unknown [5], making RPL a complex and challenging issue in reproductive medicine. The lack of a clear etiology can cause significant distress for patients, their families, and healthcare providers heightening anxiety and uncertainty.

Hypercoagulable states have also been implicated in pregnancy complications [6]. Thrombotic damage to the placenta and impaired blood flow may contribute to adverse pregnancy outcomes. Contact system including factor XII (FXII) has been suggested as a potential factor in RPL pathophysiology [7].

This review aims to examine existing literature and explore the association between the plasma concentration of coagulation FXII and RPL. Given that FXII levels can be influenced by both congenital and acquired conditions, this review also includes studies investigating the impact of the C46T polymorphism and autoantibodies to FXII, which may contribute to FXII level variations in the context of RPL.

### 1.1 FXII and Contact System–Triggered Pathways

**FIGURE 1:**
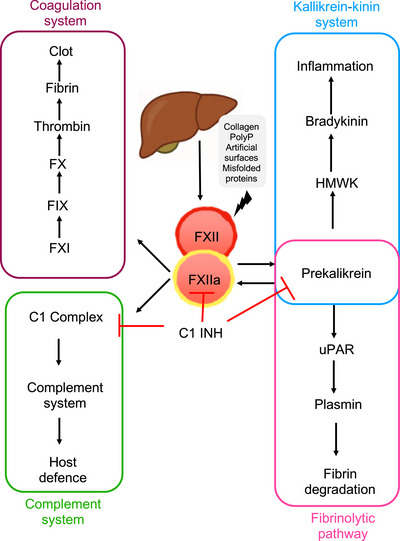
FXII and contact‐system triggered pathways. The contact system is a cascade of plasma proteins activated by FXIIa, formed when FXII encounters anionic surfaces such as polyphosphates, nucleic acids, heparin, collagen, misfolded proteins. FXIIa initiates proinflammatory and procoagulant pathways by activating prekallikrein (PK), which further amplifies FXII activation. Inflammation occurs via bradykinin release from HK, while FXIIa also activates FXI, leading to thrombin generation and clot formation. Additionally, FXIIa enhances fibrinolysis through plasmin and activates the classical complement pathway. C1 inhibitor (C1INH) regulates this system and is the main physiological inhibitor of FXIIa.

FXII is an 80 KDa serine protease produced in the liver [8]. It plays a role at least four different systems including coagulation, fibrinolysis, inflammation, and complement system [9] (Figure 1).

FXII is a key component of the intrinsic coagulation pathway and undergoes activation through proteolytic cleavage, converting it into its active form, FXIIa. This activation can occur either through autoactivation upon contact with negatively charged surfaces such as polyphosphates, nucleic acids, heparin, collagen, misfolded proteins [10], or via proteolysis mediated by enzymes such as kallikrein and plasmin [11, 12]. Once activated, FXII triggers the intrinsic coagulation pathway by activating factor XI (FXI), which subsequently activates factor IX (FIX). Together with its cofactor, factor VIII (FVIII), FIX facilitates the activation of factor X (FX), linking the intrinsic pathway to the common coagulation cascade. This ultimately leads to thrombin generation, converting fibrinogen into fibrin and forming a stable clot [13, 14]. Interestingly, FXII‐deficient mice, like humans, exhibit normal hemostasis without bleeding abnormalities. However, these mice fail to develop thrombosis in response to vessel injury. The administration of human FXII restores thrombus formation, indicating that FXII is required for pathological coagulation and thrombus growth but not for normal hemostasis [15]. However, whether the contact system contributes to FXII‐driven thrombosis in stroke models remains unclear.

Beyond coagulation, activated FXII also converts prekallikrein to kallikrein, establishing a positive feedback loop that leads to further activation of FXII. Kallikrein subsequently cleaves high molecular weight kininogen (HMWK), releasing the vasoactive proinflammatory peptide bradykinin. Bradykinin is the end product of “the contact system” [16] and plays a role in the inflammatory response [13]. The contact system has also the capacity to activate the classical pathway of the complement system and contribute to fibrinolysis [13, 17]. Bradykinin is a potent and specific stimulator of the release of endothelial cell‐derived tissue‐type plasminogen activator (tPA). Kallikrein contributes to fibrinolysis by activating single‐chain urokinase into two‐chain urokinase (prourokinase → urokinase) [13, 14]. Both tPA and urokinase activate plasminogen to plasmin, which cleaves fibrin and leads to the dissolution of the fibrin clot [14].

### 1.2 Factor XII and Pregnancy Loss

Contact system proteins, including FXII, are present in the human placenta and play a crucial role in modulating placental function. This system contributes to placental homeostasis by regulating maternal–fetal blood flow and facilitating the transplacental exchange of metabolites, thereby supporting fetal development [18]. A significant reduction in kallikrein generation, prekallikrein, and cleaved HMWK has been recently reported during pregnancies complicated by preeclampsia, suggesting that contact system dysregulation may contribute to the disease pathophysiology [19].

FXII deficiency has been proposed to influence embryonic development by affecting interactions at the maternal–fetal interface [20]. Although it has been suggested that microthrombus formation may be the underlying mechanism by which FXII deficiency contributes to pregnancy loss [20, 21], it remains unclear whether FXII deficiency directly predisposes individuals to thrombosis. The available evidence is inconclusive, as cases of both deep venous thrombosis and mild bleeding during delivery have been reported in women with FXII deficiency [22]. Additionally, abnormal placental invasion is a recognized contributor to miscarriage in thrombophilic disorders; however, no studies have specifically examined the impact of FXII deficiency on placental invasiveness [7]. Notably, previous studies have reported the prevalence of FXII deficiency up to 22% of women with RPL [23], highlighting its potential relevance in pregnancy complications.

### 1.3 The C46T Polymorphism

In the early 2000, a C→T polymorphism at nucleotide 46 in the 5′‐untranslated region of the FXII gene was identified. This variant reduces FXII translation efficiency and is associated with lower FXII levels [24]. Therefore, the FXII gene was suggested as a candidate gene for RPL. The prevalence of this variant is notably high in individuals of East Asian descent (47% vs. 10%) [24], which corresponds to their plasma Factor XII levels being approximately half of those observed in Caucasians [25].

### 1.4 Autoantibodies to FXII

The presence of antibodies targeting FXII has been suggested to reduce the availability of functional FXII, thereby impairing fibrinolytic activity and consequently contributing to a hypercoagulable state [26, 27]. This disruption is believed to compromise placental perfusion potentially leading to RPL [28].

Although FXII is a component of the coagulation pathway, it has been suggested that its primary role is in fibrinolysis rather than coagulation in reproductive medicine. Within the fibrinolytic cascade, kininogen also plays a crucial role. Consequently, kininogen‐dependent antiphosphatidylethanolamine (aPE) antibodies and/or FXII deficiency may suppress the fibrinolytic system. As a result, both aPE and anti‐FXII antibodies exhibit similar effects, not only by inhibiting fibrinolysis, but also by enhancing platelet activation, ultimately promoting a prothrombotic state [16].

## 2 Methods

The literature search and selection were done according to the Preferred Items for Systematic Reviews and Meta‐Analysis (PRISMA) [29].

### 2.1 Search Strategy

In collaboration with an experienced librarian from “Library service” at Aarhus University Hospital, the following search strategy was developed:

“(“Pregnancy Outcome”[Mesh] OR “Pregnancy, High‐Risk”[Mesh] OR “Pregnancy Complications”[Mesh] OR “pregnancy outcome*” OR “pregnancy complication*” OR “high‐risk pregnan*” OR (“abortion*”[tw] OR “recurrent pregnancy loss” OR “recurrent fetal loss” OR “recurrent foetal loss” OR “recurrent miscarriage” OR “abort*” OR “habitual abortion”) OR “fetus mortus”[tw]) AND (“Factor XII”[Mesh] OR “Factor XII”[tw] OR “Factor Twelve”[tw] OR “Hageman Factor”[tw] OR “factor 12”[tw])”.

The literature for this study was retrieved through a systematic search of the PubMed database and EMBASE. For EMBASE the abovementioned search strategy was translated with the help of “query translator” and conference and meeting abstracts were excluded.

The PubMed literature search was carried out independently by two of the authors at different times. The Embase database search was performed by one author. The literature search covered publications up to March 2025 and was supplemented by a manual review of the reference lists of the included articles.

This scoping review has been registered with the Open Science Framework (https://osf.io/b2t79)

### 2.2 Eligibility Criteria and Study Selection

Only articles written in English reporting clinical trials, observational studies, case–control studies, and cohort studies were included in this paper. Only articles published after 1970 were accepted. Inclusion criteria comprised studies investigating association between FXII and RPL, including those on C46T polymorphism and anti‐FXII antibodies due to their potential influence on FXII levels.

Exclusion criteria encompassed studies involving animal models, studies in which antiphospholipid syndrome (APS), antiphospholipid antibodies (aPL) used in APS diagnosis, or systemic lupus erythematosus (SLE) were the primary focus or the underlying condition of the study population, studies addressing pregnancy complications not related to RPL, and studies focusing on topics unrelated to pregnancy. Exclusions were applied following abstract screening and, where necessary, full‐text review.

We did not restrict study inclusion based on a specific definition of RPL, nor did we require uniform inclusion and exclusion criteria across studies.

## 3 Results

### 3.1 Study Selection

**FIGURE 2:**
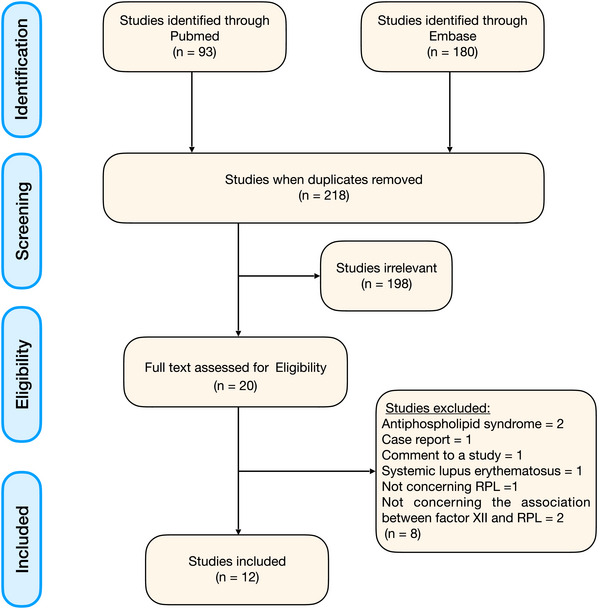
PRISMA study selection flowchart.

The search identified 218 potentially relevant studies. Of these, 20 were selected for detailed evaluation. Following full‐text assessment, 12 articles met the eligible criteria and were included in the review [4, 11, 20, 21, 23, 24, 25, 27, 30, 31, 32, 33]. See the PRISMA flowchart in Figure 2 for details.

### 3.2 Study Design, Study Population, Inclusion and Exclusion Criteria and Geography

All 12 studies included in this review were carried out between 1992 and 2015 with sample size ranging from 16 to 1257 women with RPL. The included studies comprised two prospective cohort studies [21, 31], three retrospective cohort studies [8, 24, 32], four cross sectional studies [11, 20, 23, 27] and three case control studies [4, 25, 33]. No epidemiological or randomized clinical trials were identified. Table 1 presents the baseline characteristics of the 12 included studies.

**TABLE 1:** Baseline characteristics of the 12 included studies.

Author, year	Study design	Number of participants (n)	Number of cases enrolled for analysis (n)^a^	Country	Population characteristics
Braulke et al.: 1993	Cross sectional study	43 cases 49 controls	37	Germany	**Inclusion**: Three episodes of pregnancy loss before 28th gestational week. Both nulliparous and multiparous. **Exclusion**: The finding of aPL (IgM and IgG) **Controls**: Age‐matched healthy women not using OCC
Gris et al.: 1997	Prospective cohort study	500 cases 150 controls	47	France	**Inclusion**: Three episodes of pregnancy loss before 16th gestational week. Primary abortion in childless women. **Exclusion**: Unknown. **Controls**: Age‐matched healthy mothers with no previous history of miscarriage (*n* = 100), childless women with a regular sexual life, no OCC (*n* = 50)
Yamada et al.: 2000	Cross‐sectional study	241	137	Japan	**Inclusion**: Two episodes of pregnancy loss **Exclusion**: Abnormalities in the genital tract, cervical incompetence, uterine myoma and/or endometriosis, APS, autoimmune disease, cytogenic aberration of a couple, thyroid dysfunction, diabetes mellitus ovarian dysfunction and/or luteal insufficiency and hyperprolactinemia.
Ogasawara et al.: 2001	Prospective cohort study	536	94	Japan	**Inclusion**: Two episodes of pregnancy loss in the first trimester. **Exclusion**: Uterine abnormalities and miscarriages with abnormal karyotype. aPL and Aspirin treatment, miscarriages related to an abnormal embryonal karyotype.
Iinuma et al.: 2002	Prospective case–control study	83 cases 67 controls	83^*^	Japan	**Inclusion**: Two episodes of pregnancy loss in the first trimester. **Exclusion**: Abnormal chromosomes in either partner or uterine abnormalities. History of second‐trimester miscarriage or thromboembolic events **Controls**: Women with no obstetric complications or history of miscarriage.
Pauer et al.: 2003	Prospective case–control study	162 cases 49 controls	100	Germany	**Inclusion**: Three episodes of pregnancy loss. No history of thrombophilia events. Both primary and secondary RPL **Exclusion**: Other known associations with fetal loss, not further specified. **Controls**: Age matched healthy women from blood donation center. No history of abortion or thrombophilia events. No OCC.
Walch et al.: 2005	Retrospective cohort study	212 cases 149 controls	212	Austria	**Inclusion**: Three episodes of pregnancy loss before 20th gestational week with same partner. Only white Middle‐European Caucasian women with parents of same ethnicity. **Exclusion**: Unknown. **Controls**: Postmenopausal women with at least two live births and no history of miscarriage.
Inomo et al.: 2008	Cross sectional study	197	17	Japan	**Inclusion**: Two episodes of pregnancy loss before 10th gestational week. **Exclusion**: APS
Asano et al.: 2014	Cross sectional	279 cases 100 controls	262	Japan	**Inclusion**: Two episodes of pregnancy loss. **Exclusion**: Uterine anomalies and chromosomal abnormalities in either partner. History of thromboembolic events, pre‐eclampsia or abruptio placentae. **Controls**: Fertile women with at least one child and no history of miscarriage.
Ozgu‐Erdinc et al.: 2014	Retrospective cohort study	1401	1257	Turkey	**Inclusion**: Two consecutive abortions or three abortions **Exclusion**: Pregnancy loss after week 12. Cases where it was not possible to measure FXII.
Dendrinos et al: 2014	Retrospective cohort study	100 cases 100 controls	100	Greece	**Inclusion**: Two episodes of pregnancy loss. Normal coagulation parameters. Only Caucasians and the same for partners. All cases underwent diagnostic work‐up to rule out verifiable cause of RPL. **Exclusion**: Thyroid dysfunction, glucose intolerance, renal or liver disease, uterine and genetic abnormalities, current infection or history of all type of infection. Autoimmune disease; SLE, APS, thrombotic disorders, hypertension, cardiac disease. Use of OCC or aspirin within a year before pregnancy. **Controls**: Healthy women with no history of thrombotic events or adverse pregnancy outcome and at least 1 live birth, similar socioeconomic status and non‐smokers.
Sato et al.: 2015	Prospective Case control	16 cases 28 controls	16	Japan	**Inclusion**: RPL not defined, FXII activity deficiency (<60%), positive for aPTT mixing test and positive for anti‐FXII **Exclusion**: Unknown. **Controls**: Healthy volunteers, who were age‐matched, were non‐pregnant female and had no history of miscarriage

Abbreviations: aPL, qantiphospholipid qntibodies; APS, antiphospholipid syndrom; aPTT, activated partial tromboplastin time; OCC, oral contraceptive; RPL, recurrent pregnancy loss; SLE, systemic lupus erythematosus.

^*^ The study was included because, subgroup analysis showed that among nine patients with TT variant, FXII activity did not differ significantly between those with and without aPL and no association showed between lupus anticoagulant and decreased FXII activity.

^a^ Analyses include FXII, C46T polymorphism or anti FXII antibody.

All selected studies investigated FXII and its impact and/or association with RPL, either by assessing FXII activity level [4, 8, 11, 20, 21, 23, 25, 31, 32], the C46T polymorphism [11, 24, 25] or antibodies against FXII [27, 33], two study investigated both FXII and C46T [11, 25].

Inclusion and exclusion criteria varied among studies. The number of pregnancy losses and the gestational age at loss differed, and some studies did not specify a gestational age. Dendrinos et al. [32], Asano et al. [11], Pauer et al. [4], Iinuma et al. [25], Ogasawara et al. [21], and Yamada et al. [20] explicitly excluded patients with known causes of RPL, although definitions varied. Dendrinos et al. [32], Asano et al. [11], Pauer et al. [4], and Iinuma et al. [25] also excluded patients with thrombophilia. Two studies included participants based on ethnicity [24, 32]. Furthermore, while eight studies [4, 11, 20, 21, 23, 25, 27, 32] appropriately excluded major clinical confounders, such as uterine anomalies, chromosomal abnormalities, and APS, some did not clearly report how potential confounders were addressed [8, 24, 31, 33]. Consequently, the risk of confounding cannot be fully excluded. For more explicit details about the studies inclusion and exclusion criteria see Table 1.

Geographically six studies were conducted in European countries [4, 8, 23, 24, 31, 32], while six were performed in Japan [11, 20, 21, 25, 27, 33].

Table 2 shows the RPL criteria, control groups, the FXII reference levels or cutoff values used in the included studies and the methods for measuring FXII activity, C46T polymorphism and anti‐FXII antibodies. All the studies focusing on FXII measured its activity using a FXII activity assay [4, 8, 11, 20, 21, 23, 25, 31, 32], the studies about C46T polymorphism [11, 24, 25] all used polymerase chain reaction (PCR), the two studies about anti‐FXII both measured by SDS PAGE and immunoblotting [27, 33].

**TABLE 2:** RPL criteria, control groups, FXII reference intervals and cutoff values for FXII deficiency, and measurement methods in the included studies.

Author	Miscarriage criteria	Control group	Cutoff values for FXII deficiency/Reference interval	Measurements methods
Braulke et al.:	Three or more episodes of pregnancy loss.	Age‐matched healthy women with no OCC.	<70%: deficiency Normal range: not defined	FXII activity assay
Gris et al.:	Three or more episodes of pregnancy loss.	Age‐matched healthy mothers with no previous history of miscarriage. 50 childless women with a regular sexual life, no OCC.	<65%; deficiency Normal range: 65%–135%	FXII activity assay
Yamada et al.:	Two or more episodes of pregnancy loss.	—	<50%: deficiency Normal range: 50%–150%	FXII activity assay
Ogasawara et al.:	Two or more episodes of pregnancy loss.	Women included history of with normal pregnancies.	<39%: deficiency Normal range: 39%–129%	FXII activity assay
Iinuma et al.:	Two or more episodes of pregnancy loss.	Women with no obstetric complications or history of miscarriage.	<46 %: deficiency Normal range: 46%–156%	FXII activity assay PCR (C46T)
Pauer et al.:	Three or more episodes of pregnancy loss.	Age‐matched healthy women from blood donation center. No history of abortion or thrombophilia events. No OCC.	<60%: deficiency normal range: 60%–130%	FXII activity assay
Walch et al.:	Three or more episodes of pregnancy loss.	Postmenopausal women with at least two live births and no history of miscarriage.	—	PCR (C46T)
Inomo et al.:	Two or more episodes of pregnancy loss.	—	—	SDS PAGE + Immunoblot (anti‐FXII)
Asano et al.:	Two or more episodes of pregnancy loss.	Fertile women with at least one child and no history of miscarriage. None of the patients or controls were receiving any medication or were pregnant at the time of the study.	<50%: deficiency Normal range: not defined	FXII activity assay PCR (C46T)
Ozgu‐Erdinc et al.:	Two consecutive episodes of pregnancy loss or three episodes of pregnancy loss	—	<60%: reduced activity <35%: deficiency Normal range: 60%–150%	FXII activity assay
Dendrinos et al.:	Two or more episodes of pregnancy loss.	Healthy women with no history of thrombotic events or adverse pregnancy outcome and at least one live birth.	Deficiency and normal range not defined	FXII activity assay
Sato et al.:	Not specified	Age‐matched healthy women, non‐pregnant and no history of miscarriage.	—	SDS‐PAGE + Immunoblot (anti‐FXII)

*Note:* —, not relevant/no data.

Abbreviations: OCC, oral contraceptive; PCR, polymerase chain reaction; SDS‐PAGE, C sodium dodecyl sulfate polyacrylamide gel.

Regarding RPL definitions four studies defined RPL as three abortions as the criterion for RPL [4, 23, 24, 31], while seven studies used a lower threshold including patient with two or more abortions [8, 11, 20, 21, 25, 27, 32], one study did not specify RPL criteria [33].

### 3.3 Low FXII Levels and RPL

The association between FXII and RPL reported in the included studies is summarized in Table 3. As shown, six out of nine studies measuring FXII activity concluded that reduced FXII levels were statistically significantly correlated with RPL [4, 21, 23, 25, 31, 32].

**TABLE 3:** The association between FXII/C46T polymorphism and RPL in the included studies.

Author	Number of cases enrolled for analysis	Prevalence of FXII deficiency in patients with RPL	Prevalence of C46T polymorphism in patients with RPL#	Condition associated with RPL	Significance
Braulke et al.	37	22%^a^	—	FXII	*p* = 0.002
Gris et al.:	47	9,4%	—	FXII	—
Yamada et al.:	137	5,1%	—	—	—
Ogasawara et al.:	94	16%	—	FXII	*p* = 0.02
Iinuma et al.:	83	8,3%^a^	CT: 43,4% TT: 47%	46C/T FXII	*p* = NS *p* = 0.012
Pauer et al.:	100	14,9%^b^ 12,1%^c^	—	FXII	*p* = 0.005^b^ *p* = 0.013^c^
Walch et al.:	212	—	CT: 74,3% TT: 25,7%	46C/T	*p* = 0.9
Inomo et al.:	17	—	—	__	__
Asano et al.:	262	10,3%,	CT: 53,1% TT: 38,5%	CT TT	*p* = 0.005 *p* = 0.136
Ozgu‐Erdinc et al.:	1257	7,4%	—	—	—
Dendrinos et al.:	100	15%	—	FXII	*p* = 0.016
Sato et al.:	16	—	—	__	—

^a^ Calculated value by the authors for this review (= [n of patients with FXII deficiency/N enrolled for analysis]*100).

^b^ Primary recurrent abortion.

^c^ Secondary recurrent abortion, CT, heterozygote variant of FXII C46T; NS, not significant; –, not relevant/no data; TT, homozygote variant of FXII C46T. # allele frequencies are presented.

In a study by Braulke et al. [23], eight out of 37 patients with RPL had low FXII levels, whereas none of the control group had any FXII deficiency. Additionally, FXII antigen levels were measured, revealing that six out of the eight FXII‐deficient patients also had decreased antigen levels. The FXII deficiency in patients with RPL was statistically significant compared to the control group (*p* = 0.002). The prevalence of low FXII levels was higher in patients with more than three abortions (four out of nine) compared to patients with exactly three abortions (four out of 28).

Pauer et al. [4] categorized patients into primary or secondary RPL groups. They found a statistically significant association between reduced FXII activity and primary RPL (*p* = 0.005). The association was also significant in secondary RPL (*p* = 0.013).

Ogasawara et al. [21] reported that 80% (four out of five) of patients with abnormally low FXII levels experienced miscarriages, compared to only 23.9% (11 out of 46) of those with normal FXII levels (*p* = 0.02).

The NOHA study group in France conducted the second‐largest study, including 500 women with unexplained RPL [31]. Their findings identified isolated Factor XII deficiency in 47 patients (9.4%) within the RPL group. In contrast, no deficiencies were observed in either of the two reference groups, which consisted of 100 healthy mothers and 50 childless women.

In the study conducted by Dendirinos et al. [32], 15 out of 100 women with RPL exhibited reduced FXII activity, whereas all 100 control participants had normal FXII activity. Additionally, FXII activity was significantly lower in the RPL group compared to the control group (*p* = 0.016).

Iinnuma et al. [25] reported that 10 out of 83 patients with RPL exhibited reduced FXII activity, compared to one out of 67 individuals in the control group. This difference was found to be statistically significant (*p* = 0.012)

### 3.4 FXII C46T Polymorphism and RPL

Three studies investigated the C46T polymorphism in relation to RPL [11, 24, 25]. The studies by Walch et al. [24] and Iinuma et al. [25] did not find a statistically significant association between the C46T polymorphism and RPL.

Walch et al. [24] examined 212 Caucasian women with RPL and reported a CT allele frequency of 74.3% and a TT allele frequency of 25.7% within the RPL group. These frequencies were identical to those observed in the control group (CT: 74.5%, TT: 25.7%) with no significant difference between the two groups (*p* = 0.9).

Iinuma et al. [25] investigated 83 women and found no significant difference in the frequency of the T allele or factor XII activity in CT and TT alleles in the RPL and control groups. The prevalence of FXII deficiency in the RPL group was 8.3%. However, while FXII levels were significantly associated with RPL (*p* = 0.012), the C46T polymorphism showed no significant correlation with RPL.

Asano et al. [11] reported a statistically significant higher frequency of the CT allele  in 262 women with RPL compared to controls (OR = 2.83; 95% CI: 1.37–5.85; *p* = 0.005). In contrast, the TT homozygote variant was not significantly associated with RPL (OR = 1.73; 95% CI: 0.84–3.58; *p* = 0.136), suggesting that the CT genotype, rather than TT, may represent a potential risk factor for RPL. Plasma FXII activity levels in the RPL group were comparable to those in the control group. Additionally, neither low FXII activity nor the CT genotype were found to be predictive of subsequent pregnancy outcomes in the cohort study.

### 3.5 Anti‐FXII Antibodies and RPL

A cross‐sectional study by Inomo et al. [27] analyzed blood samples from 197 women with RPL, identifying 17 women, who tested positive for anti‐FXII antibodies. The study suggested that autoantibodies to FXII inhibit FXII´s physiological function, leading to miscarriage.

Similarly, Sato et al. [33] compared 16 RPL patients with 28 healthy controls and found that all 16 RPL patients had FXII deficiency and tested positive for anti‐FXII antibodies. Furthermore, platelet aggregation was significantly more frequent in the RPL group with positive anti‐FXII antibodies than in the control group (*p* = 0.003).

## 4 Discussion

Our findings from this review indicate that reduced FXII levels and autoantibodies to FXII, but not the C46T polymorphism of FXII, appear to be significantly associated with RPL.

The potential involvement of FXII deficiency in RPL was first proposed in the late 1980s [34], prompting studies on the topic between the 1990s and 2015. However, no comprehensive review article has been published on this subject, nor have any new studies emerged since then that would meet the eligibility criteria for inclusion in the present review.

Although FXII has an impact on both the coagulation and fibrinolytic pathways, the precise mechanism by which it may contribute to RPL remains unclear. The fibrinolytic system plays an important role during early trophoblast invasion, and FXII deficiency, which negatively affects fibrinolysis, may impair trophoblast invasion [26]. Furthermore, it has been hypothesized that FXII deficiency may lead to the formation of microthrombi at the maternal–fetal interface, thereby impairing adequate fetal growth [7]. However, it is important to note that Iwaki et al. [35] reported that female mice homozygote for total FXII deficiency had normal pregnancies and litter sizes, suggesting that congenital FXII deficiency may not be directly associated with pregnancy loss. Despite this, the present review identified a higher prevalence of reduced FXII activity in women with RPL.

Two major inconsistencies persist across studies, as outlined in Table 2. One of the key inconsistencies is the definition of RPL across studies, the included studies did not adhere to a uniform miscarriage criterion, which may influence the comparability of findings. Braulke et al. [23] suggested that FXII deficiency increases with the number of miscarriages.

Among studies using ≥2 miscarriages as the RPL definition, three out of seven reported a significant association between FXII deficiency and RPL [21, 25, 32]. Among studies using ≥3 miscarriages, three out of four reported statistically significant findings [4, 23, 31].

The second major lack of consensus in the studies is the reference interval of FXII used and the cutoff for FXII deficiency. Ozgu‐Erdinc et al. [8] and Pauer et al. [4] defined the normal reference interval to be between 60%–130% and 60%–150% respectively. Whereas Ogasawara et al. [21], Gris et al. [31], and Iinuma et al. [25] established their own reference intervals from healthy controls, reporting 39%–129%, 65%–135%, and 46%–156% respectively, some studies did not specify their reference range for FXII [11, 23, 32]. It is important to note that the FXII reference intervals have been established using non‐pregnant individuals, and since most clotting factors increase during pregnancy, it remains unknown how FXII levels fluctuate during pregnancy, introducing a potential bias in these studies.

Notably, there was no consensus on FXII deficiency either. Two studies [11, 20] used <50% as the cutoff value, two used <60% [4, 8] while others used varying cutoff values such as: <70% [23], <65% [31], and <39% [21] as presented in Table 2. Additionally, Ozgu‐Erdinc et al. [8] defined actual deficiency as <35% and <60% as reduced FXII activity level. The variability in cutoff values for FXII deficiency (e.g., 35%–70% activity) suggests that no consensus exists for determining on when FXII activity is low enough to be considered pathological in RPL. Furthermore, many individuals with low FXII levels do not experience RPL. This suggests that FXII deficiency alone may be insufficient to cause pregnancy loss but may act alongside other prothrombotic or immunologic factors. Given these uncertainties, routine FXII screening in RPL is not currently recommended by guidelines. Further prospective studies are needed to clarify its clinical significance.

The prevalence of FXII deficiency in RPL patients across studies ranged from 5.1% to 22% (see Table 3), likely due to differences in FXII reference intervals used in previous studies, genetic variation, or population ethnicity. For example, the frequency of the FXII C46T polymorphism, which is associated with lower plasma FXII levels, was substantially higher in Japanese population compared to Caucasians (47% vs. 10% homozygote mutant genotypes) [24]. However, in this review, studies involving Japanese patients did not demonstrate a higher prevalence of FXII deficiency compared to other populations (see Table 3). FXII deficiency is a relatively rare condition, and the sample size of RPL patients with FXII deficiency was small. Study cohorts varied significantly, ranging from the largest cohort of 1257 cases [8] to the smallest with only 16 cases [33]. Yamada et al. [20]. investigated the prevalence of FXII deficiency in a population of 241 women with RPL, and of those 137 had RPL of unknown cause. Only seven women (5.1%) had FXII deficiency, and two of these patients also had elevated natural killer (NK) cell activity, a known risk factor for adverse pregnancy outcomes, which could be a potential confounding factor. Ogasawara et al. [21]. conducted a prospective cohort study investigating 536 women with RPL, but FXII activity was measured in only 94 cases, making the remaining 442 patients irrelevant for this review. Among the women with FXII deficiency (*n* = 5), four had experienced RPL (80%), but the sample size was too small to draw definitive conclusions.

Research on homozygote FXII‐deficient mice has found no association between FXII deficiency and pregnancy loss, suggesting that anti‐FXII antibodies, rather than FXII deficiency, may play a role in RPL [35]. Two studies [27, 35] included in this review investigated the presence of anti‐FXII antibodies in RPL and found that these antibodies may disrupt FXII's physiological function, potentially contributing to pregnancy loss. However, the small sample sizes in both studies limit the strength and reliability of these findings, emphasizing the need for further large‐scale research to validate these associations.

Several studies used different control groups, which may introduce bias. Dendrinos et al. [32]. compared 100 women with RPL to 100 healthy women with no history of thrombotic events or adverse pregnancy outcome and at least one live birth. The study showed that 15 of the women with RPL had FXII deficiency. Pauer et al. [4] found an association between FXII deficiency and RPL by investigating 100 women with RPL with no history of thrombotic events and comparing them to a group of 49 age‐matched healthy women from a blood donation center, who had no history of abortion or thrombotic events and did not use oral contraceptives (OCC). Gris et al. [31]. investigated 500 women with RPL and used both a group of age‐matched healthy mothers with no previous history of miscarriage and a group of childless women with a regular sexual life and no OCC. None of the women from the two reference groups had FXII deficiency. In contrast to the aforementioned studies, Walch et al. [24]. used postmenopausal women with at least two live births and no history of miscarriage as the control group, comparing them to 212 women with RPL. The authors stated that this approach was used to eliminate the risk of future miscarriages in the control group, which could otherwise introduce bias if one of the controls experienced a miscarriage after the study's completion. In line with this concern, there is a risk of bias in most of the included studies, as they cannot entirely exclude the possibility that control participants may later experience miscarriage.

Of the three studies examining the C46T polymorphism [11, 24, 25], only one found an association with RPL. Walch et al. [24] and Iinuma et al. [25] reported that the C46Tpolymorphism frequency was similar in RPL women and controls, but low FXII levels were associated with RPL. Asano et al. [11] found that FXII plasma activity was similar in both groups, but there was a higher frequency of the CT polymorphism in RPL women. Since only one of the three studies [11] found a significant association, this suggests that the C46T polymorphism may not be the right target in relation to RPL. It also raises the question of whether the C46T polymorphism is related to FXII levels or could be an independent risk factor for RPL.

Sato et al. [33] reported that six out of 16 RPL cases with FXII deficiency also tested positive for aPE antibodies. The inclusion of aPE‐positive women in the study may have influenced the observed association between FXII deficiency and RPL. It is also important to note that the presence of aPL can reduce FXII activity and may also contribute to RPL [12, 28, 34, 36]. Jones et al. [28] reported that FXII antibodies were significantly associated with fetal loss in patients with APS (*p* = 0.025). Another study from Jones et al. [37] reported that FXII antibodies were common in lupus anticoagulant (LA)‐positive patients, leading to a misdiagnosis of FXII deficiency. This raises the possibility that FXII deficiency is not the primary cause of RPL, but rather a secondary effect of LA or APS, though this hypothesis requires further investigation.

A recent study by Morita et al. [38] involving pregnant Japanese women with a history of RPL found a prevalence of FXII deficiency of 7.6% (102/1340) and the prevalence of aPL 8.7% (116/1340) within the same cohort. Given the potential interaction between aPL and FXII, which may lead to reduced FXII levels, this study was excluded from the current review to avoid confounding effects. The study of Morita et al. [38] compared live birth rates among FXII‐deficient women who received no treatment, low‐dose aspirin alone, or a combination of low‐dose aspirin and unfractionated heparin during pregnancy. The live birth rate was statistically higher in both treatment groups compared to the no‐treatment group (Low‐dose aspirin: 63.8%, low‐dose aspirin combined with unfractionated heparin: 70.8%, no treatment: 27.3%; *p* = 0.028 and *p* = 0.016, respectively). However, there was no significant difference in live birth rate between the two treatment groups (*p* = 0.555).

As demonstrated in this review, low FXII levels can be present in patients with unexplained RPL even in the absence of APS. Although current guidelines do not recommend genetic thrombophilia testing in the evaluation of unexplained RPL [39], FXII measurement may be considered in selected cases where no other cause is identified. Based on the study by Morita et al. [38], off‐label use of low‐dose aspirin or a combination of low‐dose aspirin and heparin may be considered in subsequent pregnancies for women with confirmed FXII deficiency. However, as this treatment approach is not widely adopted outside of Japan, further evidence from prospective studies is needed before FXII testing or targeted therapy can be recommended as part of routine RPL management.

### 4.1 Strengths and Limitations

A key strength of this study is its novelty, as it represents the first comprehensive review to systematically re‐evaluate the available data on the association between FXII levels and RPL using a systematic, transparent, and stringent approach. Notably, based on the inclusion and exclusion criteria of this review the most recent study on this topic meeting eligibility was published in 2015, suggesting a decline in research activity in this area. This can be seen as a limitation raising concerns about the currency of the data, but also a noteworthy finding highlighted by our review. The absence of studies from the past decade does not negate the potential relevance of earlier findings. We believe that revisiting older studies with an updated perspective is both timely and relevant, reinitiating discussions concerning the potential clinical significance of contact activation in RPL. Furthermore, heterogeneity among the included studies poses challenges for direct comparisons and limits the ability to draw generalizable conclusions. Additionally, most studies originate from two geographic regions—Japan and Europe—which may introduce population bias, limiting the applicability of findings to broader demographics. Thus, aPL and APS can interfere with FXII, leading to reduced FXII levels. Studies that did not investigate aPL/APS or did not use them as an exclusion criterion may carry a risk of misinterpretation, potentially impacting the conclusions of this review. Furthermore, as is typical for scoping reviews, this paper did not include systematic assessment of the risk of bias or the quality of included studies, which may limit the critical evaluation of findings. Nevertheless, it is worth noting that most included studies had small sample sizes, and only half controlled for confounders, resulting in an overall moderate to low methodological quality. Additionally, the exclusion of non‐English publications may have introduced language bias, further limiting the scope of available evidence.

## 5 Conclusion

This scoping review highlights the evidence supporting an association between FXII deficiency, autoantibodies to FXII and RPL, while the relationships between the C46T genetic polymorphism and RPL remain inconclusive. In recent years, the contact system has attracted increasing interest in medicine, with expanded parameters and novel analytical methods. Given its potential relevance, revisiting the role of contact activation protein, FXII in the evaluation of RPL patients may provide valuable clinical insights. Finally, well‐powered studies are essential to clarify the significance of the contact system, including FXII activity and autoantibodies to FXII, in RPL pathophysiology. Importantly, the diagnosis of FXII deficiency may be misleading in the presence of aPL/APS, further emphasizing the need for careful interpretation in future research.

## Ethics Statement

It was not required due to this is a review.

## Conflicts of Interest

No conflicts of interest.

## Data Availability

This scoping review is based on previously published studies. All data supporting the conclusions are available within the cited literature.
